# ErbB1 epidermal growth factor receptor is a valid target for reducing the effects of multiple inhibitors of axonal regeneration

**DOI:** 10.1016/j.expneurol.2012.09.007

**Published:** 2013-01

**Authors:** Veronica H.L. Leinster, Mary T. Joy, Raisa E. Vuononvirta, Stephen R. Bolsover, Patrick N. Anderson

**Affiliations:** a Research Department of Cell and Developmental Biology, UK; b Research Department of Oncology, University College London, UK

**Keywords:** CNS, Central nervous system, CSPG, Chondroitin sulfate proteoglycans, DRG, Dorsal root ganglia, EGF, Epidermal growth factor, TLR3, Toll-like receptor 3, ErbB1, Axon regeneration, Epidermal growth factor receptor, Neurite outgrowth, Spinal cord injury, CNS myelin, Fibrinogen, Polyinosinic:polycytidylic acid, Chondroitin sulfate proteoglycans

## Abstract

Pharmacological inhibitors of epidermal growth factor receptor (ErbB1) attenuate the ability of CNS myelin to inhibit axonal regeneration. However, it has been claimed that such effects are mediated by off-target interactions. We have tested the role of ErbB1 in axonal regeneration by culturing neurons from ErbB1 knockout mice in the presence of various inhibitors of axonal regeneration: CNS myelin, chondroitin sulfate proteoglycans (CSPG), fibrinogen or polyinosinic:polycytidylic acid (poly I:C). We confirmed that ErbB1 was activated in cultures of cerebellar granule cells exposed to inhibitors of axonal regeneration and that ErbB1 kinase inhibitors promoted neurite outgrowth under these conditions. In the presence of myelin, fibrinogen, CSPG and poly I:C ErbB1 −/− neurons grew longer neurites than neurons expressing ErbB1. Furthermore, inhibitors of ErbB1 kinase did not improve neurite outgrowth from ErbB1 −/− neurons, ruling out an off-target mechanism of action. ErbB1 kinase activity is therefore a valid target for promoting axonal elongation in the presence of many of the molecules believed to contribute to the failure of axonal regeneration in the injured CNS.

## Highlights

► We test the role of ErbB1 EGFR receptor in controlling neurite outgrowth. ► CNS myelin, CSPGs, fibrinogen and Poly I:C inhibited neurite outgrowth. ► Outgrowth from wild type neurons was disinhibited by ErbB1 kinase inhibitors. ► Disinhibition was shown to be dependent on the presence of ErbB1. ► ErbB1 kinase is a valid target for enhancing axonal regeneration in the CNS.

## Introduction

Axons do not normally regenerate in the adult mammalian CNS. Conditions such as complete spinal cord injuries therefore result in permanent and very serious functional deficits. There are many potent inhibitors of axonal regeneration in the injured CNS including myelin-associated proteins, CSPG, fibrinogen and axonal guidance molecules. These are believed to combine to make the damaged spinal cord very unfavourable for axon regrowth (Anderson et al., 2007; Bolsover et al., 2008; Busch and Silver, 2007; Sandvig et al., 2004; Tang, 2003; Verma and Fawcett, 2005; Wehrle et al., 2005). The EGF receptor ErbB1 may play an important role in regulating axonal regeneration. In some studies, stimuli that increase ErbB1 phosphorylation have been shown to promote axon outgrowth (Evangelopoulos et al., 2009; Goldshmit et al., 2004; Tsai et al., 2010). However, other studies indicate that ErbB1 may be a critical mediator of outgrowth-inhibitory cues in the adult CNS. Exposure to Nogo-66 or OMgp (inhibitory components of CNS myelin) or CSPG, causes phosphorylation of ErbB1 in an intracellular calcium-dependent manner (Koprivica et al., 2005). Inhibitors of ErbB1 kinase activity allow axons to grow over an inhibitory substrate of myelin or CSPG *in vitro* and through a crush injury of the optic nerve *in vivo* (Koprivica et al., 2005). Fibrinogen, which enters CNS lesions after trauma, acts through β3 integrin to cause ErbB1 phosphorylation; axon outgrowth is inhibited but can be rescued by application of an ErbB1 kinase inhibitor (Schachtrup et al., 2007). Axon outgrowth over fibroblasts is enhanced by treatment with ErbB1 inhibitors (Povlsen et al., 2008). Inhibiting ErbB1 kinase activity greatly enhanced axonal regeneration through a crush injury of the mouse optic nerve *in vivo* (Koprivica et al., 2005) and it has been reported that treatment with an ErbB1 kinase inhibitor enhanced functional recovery following spinal injury in rats (Erschbamer et al., 2007). However, an attempt at replication of the latter finding on spinal injury was not successful (Sharp et al., 2012). These results therefore suggest a model in which a large number of clinically important inhibitors of CNS axonal regeneration activate ErbB1, and the activated ErbB1 in some way acts to reduce or even eliminate axon outgrowth or regeneration. Since the inhibitors of ErbB1 that have been shown to enhance axonal regeneration include the licensed drug Erlotinib, these observations have potentially important clinical applications.

However, experiments using siRNA to knock down ErbB1 expression *in vitro* have yielded results inconsistent with this growing consensus. Cultures in which ErbB1 expression had been dramatically reduced by treatment with siRNA showed undiminished inhibition of axon outgrowth by myelin, and the ErbB1 kinase inhibitor AG1478 retained its ability to rescue axon outgrowth. On the basis of this and other evidence it was suggested that AG1478 exerted its axon-promoting effect through an action on a protein other than ErbB1 (Ahmed et al., 2009; Douglas et al., 2009). However, siRNA rarely eliminates the target protein completely. We therefore re-examined this question by using neurons from ErbB1 knockout mice in which the protein is completely absent. If PD168393 and AG1478 attenuate the effects of inhibitors of CNS axonal regeneration in these neurons, then they would be certainly acting off-target. However, we saw no such protection. Rather, our results confirm the central role of ErbB1 in mediating the inhibition.

In addition we sought to examine whether the nucleic acids can also inhibit axonal growth through ErbB1. Double stranded RNA and its analogue poly I:C, acting upon Toll-like receptor 3 (TLR3), have been reported to inhibit axon outgrowth from sensory neurons (Cameron et al., 2007). TLR3 may be activated by RNA released from damaged mammalian cells (Kariko et al., 2005), or by viral RNA. We asked whether this dramatically different cue also operated through ErbB1 and whether this effect, like that of CNS myelin, involved changes of intracellular calcium.

## Materials and methods

**Supplementary Fig. 1.**
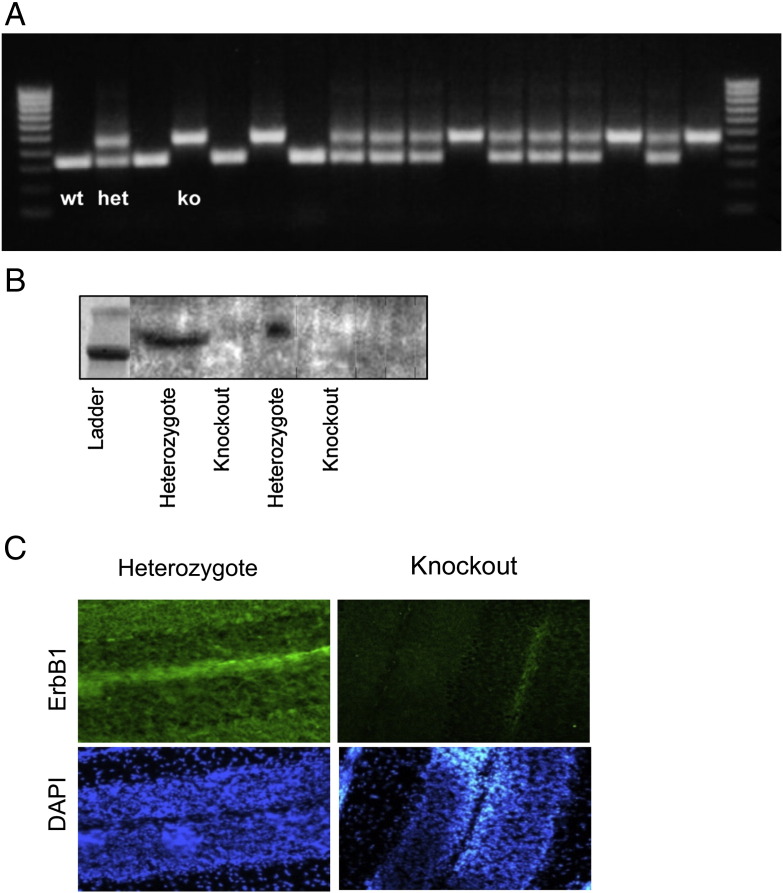
A: Typical PCR product run after genotyping of wild type, heterozygote and ErbB1 −/− animals. Ladder is 100, 200, 300 bp etc.; the wild type gene generates a 350 bp product whilst the modified, non-productive gene generates a 450 bp product. B: Western blot of proteins from E18.5 embryos identified by PCR as heterozygote or ErbB1 −/−. Ladder lane shows bands at 200 and 150 kD. C: Coronal cerebellum sections from P14 pups. Immunolabelling using anti-ErbB1 shows expression in Purkinje cells, granule cells and subcortical white matter of control mice but only background signal in −/− mice.

ErbB1 +/− mice were obtained from the Jackson Labs (Strain *Egfr*^*tm1Mag*^*/J*) and maintained in a CD1/MF1 mixed outbred colony. Genotyping used primers and PCR conditions suggested by Jackson Labs, specifically the common primer 5′GCCCTGCCTTTCCCACCATA3 ′, the mutant-specific primer 5′TTGCAGCACATCCCCCTTTC3 ′ generating a 450 bp amplicon and the wild type specific primer 5′ATCAACTTTGGGAGCCACAC3′ generating a 350 bp amplicon. Genotyping was performed in the UCL Sequencing and Genotyping Facility and gave unequivocal results (Supplementary Fig. 1A). ErbB1 −/− pups were recognised at birth by their open eyes and at later days by lack of hair. All pups used for dissection were genotyped to confirm identification. Western blots of brains dissected from E18.5 embryos identified as ErbB1 −/− confirmed the absence of the protein (Supplementary Fig. 1B).

To culture cerebellar granule neurons (CGNs), cerebella were dissected from humanely killed P5–P7 pups and minced manually followed by digestion with 0.05% trypsin for 12 min at 37 °C in phosphate buffered saline. Digestion was terminated by adding excess foetal bovine serum. The suspension was centrifuged and the pellet resuspended in 2000 units/ml DNAase (Invitrogen) in Neurobasal medium (Invitrogen). After trituration large pieces were allowed to settle and removed; the suspension was then centrifuged and the pellet resuspended in growth medium comprising Neurobasal medium plus 2% B27 supplement, 0.4 mM additional glucose, 20 mM additional KCl, 20 μM glutamine, 100 units/ml penicillin and 100 μg/ml steptomycin followed by plating in 24 well plates at 65,000 cells per well.

Sensory neuron cultures used a growth medium of F12 with Glutamax (Invitrogen) supplemented with 100 units/ml penicillin and 100 μg/ml streptomycin and either 10% foetal calf serum or, where noted, 1% N2 supplement (Invitrogen). Dorsal root ganglia from P7–P9 pups were dissected and incubated in a mixture of collagenase (5 mg/ml), dispase (2 mg/ml) and DNAase (1000 U/ml) for 40 min at 37 °C. The enzyme reaction was stopped by addition of excess growth medium. The cells were then centrifuged for 10 min at 1000 rpm. After centrifugation, the supernatant was discarded and 1 ml of growth medium was added, triturated and centrifuged for 5 min at 400 rpm. The resultant supernatant was discarded and 1 ml of growth media was added and triturated to form the cell suspension. In order to reduce the number of non-neuronal cells, a BSA cushion (15%) was prepared in growth medium. The cell suspension was slowly added through the sides of the tube such that the suspension rested on the BSA solution. It was then centrifuged at 1000 rpm for 10 min. The neuronal cells formed the pellet at the bottom of the tube and the non-neuronal cells were seen as a white layer at the interface. The pellet was then resuspended in growth medium and plated in eight chambered glass slides (VWR).

Substrates were primed by treatment with poly-l-lysine (Sigma, 100 μg/ml overnight). An inhibitory myelin substrate for sensory neuron culture was prepared by incubating primed slides for 4 h with myelin (100 μg/ml) followed by incubation for 2 h with 2 μg/ml laminin. In all other preparations, primed substrates were incubated for 4 h with laminin (10 μg/ml for CGNs, 2 μg/ml for sensory neurons) mixed where appropriate with myelin or CSPG (100 μg/ml and 5 μg/ml respectively). In contrast the inhibitory agents fibrinogen and poly I:C were added to the growth medium.

Cerebellar granule cell cultures were fixed with 4% paraformaldehyde after 16 h, whilst sensory neurons were fixed after 24 h (48 h for cultures on myelin). Cultures were stained for neuron-specific β3 tubulin (rabbit polyclonal, Sigma) and with Hoechst 33342 (Sigma). Images were acquired using a Zeiss Axiophot microscope, Hamamatsu C4742 camera and Improvision software. The total neurite length for each imaged neuron was measured using the Neurite Tracer plug-in for the ImageJ software package. For cerebellar granule cells, neurite lengths on one individual cover slip (10 images at 20 × magnification) were averaged to yield a single value. In most experiments statistical assessment was performed on measurements obtained from 2 coverslips from each of 3 animals (N = 6). For sensory neurons, neurite lengths from all four wells with the same culture conditions, comprising measurements of more than 30 sensory neurons, were averaged to yield a single value from each animal.

Data from each experiment were analyzed by ANOVA followed by a set of post tests using Bonferroni correction. These comprised three types of comparison. First, for each culture environment, neurite outgrowth from ErbB1 knockout neurons was compared with outgrowth from heterozygote neurons under the same culture conditions. Second, neurite outgrowth in experimental conditions was compared with outgrowth from neurons of the same genotype under control conditions. Lastly to assess whether a drug treatment gave significant rescue of neurite outgrowth, neurite length in the presence of a neurite outgrowth inhibitor and additional drug was compared with neurite length of the same genetic type with the same outgrowth inhibitor but without the drug.

Phosphorylation of ErbB1 in cerebellar granule cells was assessed by scraping cells off the substrate, washing twice with phosphate buffered saline, and lysing for 45 min at 4 °C in CelLytic lysis buffer (Sigma Aldrich) supplemented with 1% protease inhibitor (Sigma) and 1%phosphatase inhibitor (Active Motif). Total protein extracts (10 μg/lane) were separated electrophoretically in 7% Tris-Acetate gel and transferred to an Immobilon membrane (Millipore). Immunodetection used rabbit primary antibodies against total and Y1068 phosphorylated EGFR (1:1000, Cell Signaling, Beverly, Massachussetts) together with monoclonal anti-tubulin (1:3000, Sigma). Finally, the primary antibodies were probed with horseradish peroxidase conjugated polyclonal goat antibodies (Cell Signaling) for chemiluminescence detection with the ECL System (GE Healthcare).

ErbB1 inhibitors PD168393 and AG1478 were obtained from Merck. BAPTA-AM and BCECF-AM were from Invitrogen.

## Results

### ErbB1 phosphorylation in the presence of the inhibitors of axonal regeneration, CNS myelin or fibrinogen

**Fig. 1:**
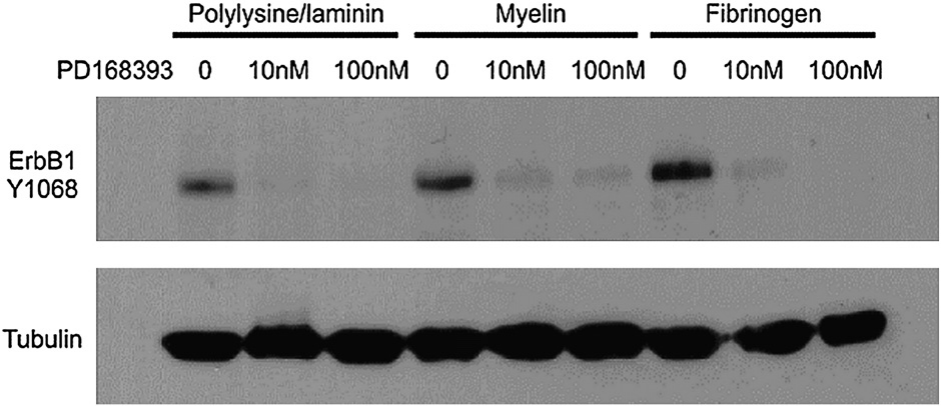
Tyrosine 1068 phosphorylation in ErbB1. Cerebellar granule cells were grown on the indicated substrates with and without PD168393 at the indicated concentrations and harvested after 16 h. Transfer membranes were probed with an antibody specific for ErbB1 phosphorylated on Y1068. ErbB1 kinase activity was increased by the presence of CNS myelin or fibrinogen but was minimal when PD168393 was added.

First we confirmed that CNS myelin and fibrinogen, potent inhibitors of axonal regeneration, elevated levels of ErbB1 phosphorylation. Phosphorylation of ErbB1 was detectable in cerebellar granule neurons on a permissive polylysine/laminin substrate, but was markedly increased in cells cultured in the presence of fibrinogen or cultured on CNS myelin. As expected, under all conditions, the ErbB1 kinase inhibitor PD168393 at 10 nM or 100 nM reduced ErbB1 activation to trace levels (Fig. 1). These findings confirm the previous observations of the acute activation of ErbB1 by myelin proteins and fibrinogen (Koprivica et al., 2005; Schachtrup et al., 2007) and show that such activation persists during many hours of culture.

### Both inhibition of ErbB1 and its genetic deletion enhance axonal regeneration in the presence of CNS myelin, chondroitin sulphate proteoglycans (CSPG), and fibrinogen

**Fig. 2:**
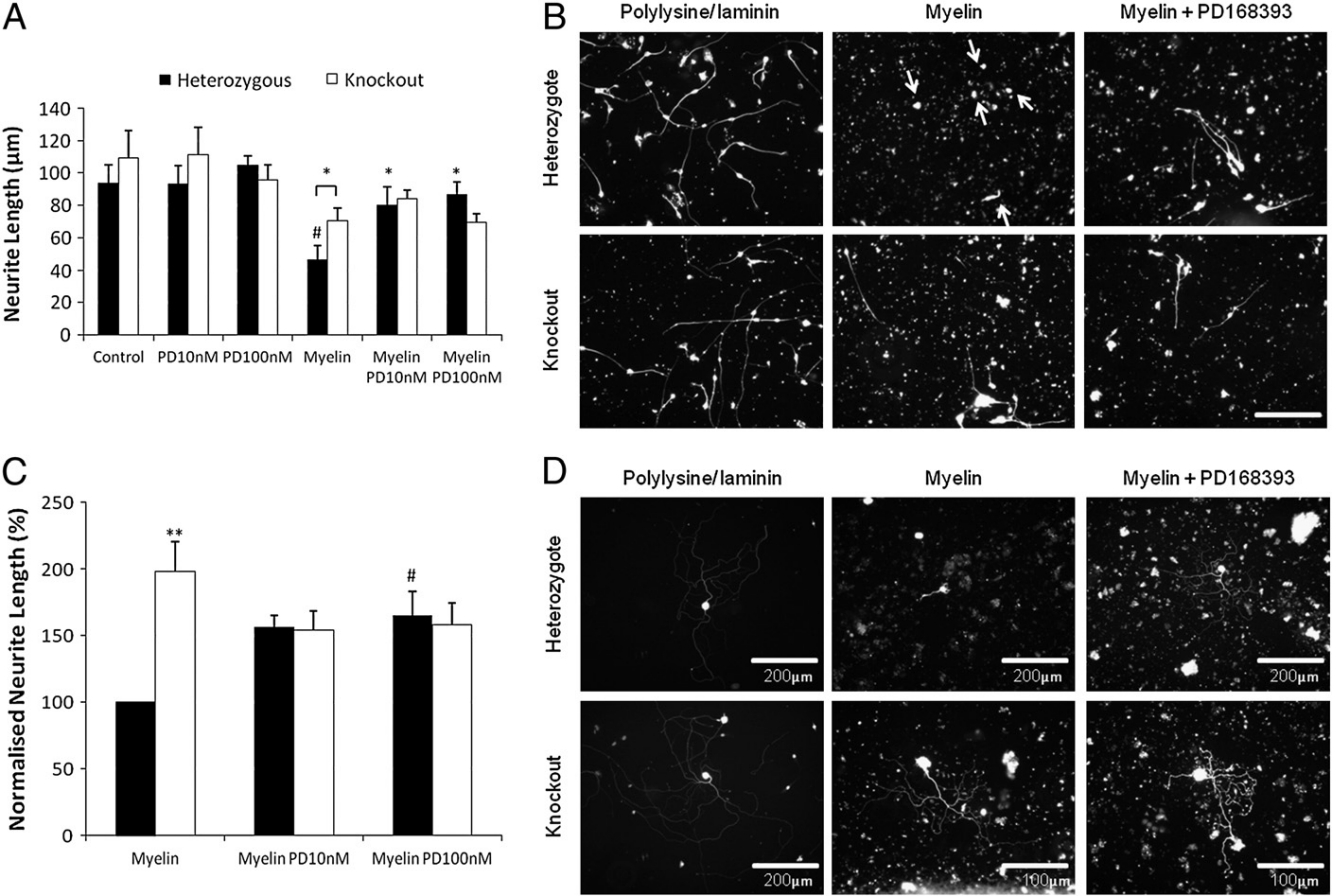
PD168393 rescues outgrowth on myelin from ErbB1-expressing neurons but does not affect ErbB1 −/− neurons. A: Heterozygous and ErbB1 −/− cerebellar granule cells were cultured on a control substrate or on a myelin substrate, in the presence of PD168393 at the indicated concentrations. N = 6. Two way ANOVA *post hoc* Bonferroni, * = p < 0.05 when compared to heterozygous cells on myelin unless shown otherwise, ^#^ = p < 0.001 when compared to heterozygous cells on a control substrate. B: Representative images of cultured cerebellar granule neurons from an ErbB1 −/− pup and heterozygote littermates on polylysine/laminin substrates with or without myelin fixed and stained for neuron-specific β3 tubulin. Neurons are indicated with arrows in the top middle panel where they would otherwise be difficult to distinguish from the fluorescent myelin fragments. The ErbB1 kinase inhibitor PD168393 was present at 10 nM where indicated. Images were taken at × 20 magnification and scale bar equals 100 μm. The scale bar applies to all panels. C: Heterozygous and ErbB1 −/− sensory neurons were cultured on a myelin substrate in the presence of PD168393 at the indicated concentrations. Neurite length was normalised to the measurement for heterozygous cells on myelin. N = 7. Two way ANOVA *post hoc* Bonferroni ** = p < 0.01 when compared to heterozygous cells under the same culture conditions, # = p < 0.05 when compared to heterozygous cells cultured on myelin. D: Representative images of cultured sensory neurons from an ErbB1 −/− pup and heterozygote littermates on polylysine/laminin substrates with or without myelin fixed and stained for neuron-specific β3 tubulin. The ErbB1 kinase inhibitor PD168393 was present at 10 nM where indicated. Note the different scales in two of the panels.

To investigate the role of ErbB1 in regulating neurite outgrowth in the absence of inhibitors of axonal regeneration we plated cerebellar granule neurons onto a substrate of polylysine plus laminin. Under these outgrowth-permissive conditions, we found no consistent effect on neurite outgrowth by the absence of ErbB1 or by treatment with its inhibitor PD168393 (Fig. 2A). CNS myelin is known to inhibit neurite outgrowth from many types of neurons (Caroni and Schwab, 1988; Chivatakarn et al., 2007; McKerracher et al., 1994). PD168393 was reported to rescue neurite outgrowth from retinal ganglion cells and DRG neurons in the presence of myelin (Ahmed et al., 2009; Koprivica et al., 2005). We confirmed that rat CNS myelin prepared in our laboratory inhibited neurite outgrowth and that this effect was relieved by PD168393. For both cerebellar granule neurons and primary sensory neurons, myelin dramatically inhibited neurite outgrowth from ErbB1-expressing cells and this inhibition was partially rescued by PD168393 at 10 nM or 100 nM (Fig. 2). Neurite outgrowth from ErbB1 −/− cerebellar granule neurons was inhibited by myelin, but to a significantly smaller extent than seen with ErbB1-expressing neurons, and the residual inhibition was not rescued by PD168393 (open bars in Fig. 2A).

Since different neurons use different receptors to detect inhibitors of neurite outgrowth (Giger et al., 2008) we also studied neurite outgrowth from ErbB1-expressing primary sensory (DRG) neurons. Neurites of ErbB1 −/− sensory neurons grown on a myelin substrate were significantly longer than were neurites of ErbB1-expressing neurons, although not as extensive as when myelin was absent, and PD168393 did not increase the neurite length of ErbB1 −/− neurites on this substrate (Figs. 2C, D — note change of scale in some panels). Hence the absence of ErbB1 or its inhibition by PD168393 significantly disinhibits neurite outgrowth from both cerebellar granule cells and primary sensory neurons cultured on CNS myelin. It has been claimed that the effects of ErbB1 inhibitors on axonal outgrowth in the presence of myelin are off-target, *i.e.* mediated by effects on a target other than ErbB1. If that hypothesis were correct then PD168393 should enhance neurite outgrowth on CNS myelin even in ErbB1 −/− cultures. In fact, neurite outgrowth from ErbB1 −/− cerebellar granule cells and DRG neurons on CNS myelin was not improved by PD168393 (open bars in Figs. 2A, C).

**Fig. 3:**
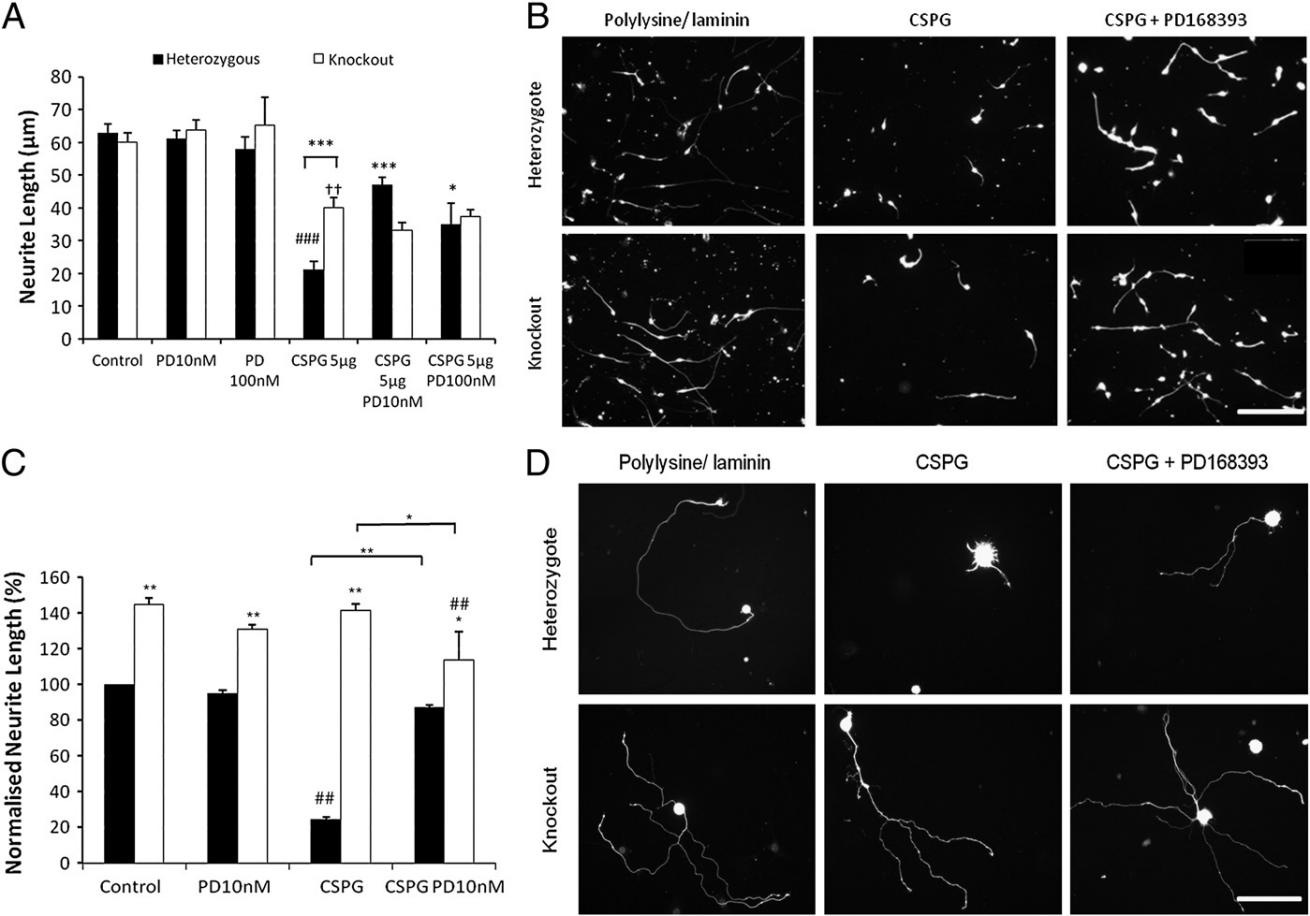
PD168393 rescues outgrowth from ErbB1-expressing neurons in the presence of CSPG but does not affect ErbB1 −/− neurons. A: Cerebellar granule cells were cultured on a control substrate or on a CSPG substrate, in the presence of PD168393 at the indicated concentrations. N = 6 Two way ANOVA *post hoc* Bonferroni, * = p < 0.05 when compared to heterozygous cells on CSPG, *** = p < 0.001 when compared to heterozygous cells on CSPG unless shown otherwise, ^# # #^ = p < 0.001 when compared to heterozygous cells on control substrate, ^††^ = p < 0.01 when compared to ErbB1 −/− cells on control substrate. B: Representative images of cultured cerebellar granule neurons from an ErbB1 −/− pup and heterozygote littermates on polylysine/laminin substrates with or without 5 μg/ml CSPG fixed and stained for neuron-specific β3 tubulin. The ErbB1 kinase inhibitor PD168393 was present at 10 nM where indicated. Images were taken at × 20 magnification and scale bar equals 100 μm. The scale bar applies to all panels. C: Sensory neurons were cultured on a control substrate or on a CSPG substrate, in the presence of PD168393 at the indicated concentrations. Neurite length was normalised to the control heterozygous measurement. N = 7. Two way ANOVA *post hoc* Bonferroni ** = p < 0.01 and * = p < 0.05 when compared to heterozygous cells under the same culture conditions unless shown otherwise. ^# #^ = p < 0.01 when compared to the control of the same genetic background. PD168393 rescues outgrowth from ErbB1-expressing neurons in the presence of CSPG but does not affect ErbB1 −/− neurons. D. Representative images of cultured sensory neurons from an ErbB1 −/− pup and heterozygote littermates on polylysine/laminin substrates with or without 5 μg/ml CSPG fixed and stained for neuron-specific β3 tubulin. The ErbB1 kinase inhibitor PD168393 was present at 10 nM where indicated. Images were taken at × 20 magnification and scale bar equals 100 μm. The scale bar applies to all panels.

CSPG are extracellular matrix and cell surface proteins known to markedly inhibit neurite outgrowth from a wide range of neurons (Fitch and Silver, 2008; Snow et al., 1990), and PD168393 was reported to rescue neurite outgrowth from retinal ganglion cells in the presence of CSPG (Koprivica et al., 2005). CSPG dramatically inhibited neurite outgrowth from ErbB1-expressing cerebellar granule cells and this inhibition was partially rescued by PD168393 at 10 nM or 100 nM (solid bars in Fig. 3A). Neurite outgrowth from ErbB1 −/− neurons was inhibited by CSPG, but to a significantly smaller extent than seen with ErbB1-expressing neurons (Fig. 3B), and the residual inhibition was not rescued by PD168393 (hollow bars in Fig. 3A).

We also studied neurite outgrowth from ErbB1-expressing primary sensory (DRG) neurons. As expected, outgrowth was dramatically inhibited by the inclusion of CSPG in the substrate (Fig. 3D). For this neuronal type, PD168393 eliminated the inhibition (solid bars in Fig. 3C). In contrast, neurite outgrowth from ErbB1 −/− DRG neurons was not inhibited at all by CSPG at the concentration we used, and PD168393 did not increase their length (open bars in Fig. 3C). Hence, the absence of ErbB1 or the presence of the ErbB1 kinase inhibitor PD168393 disinhibits neurite outgrowth from both cerebellar granule cells and sensory neurons grown on CSPG. If the effects of ErbB1 inhibitors on axonal outgrowth in the presence of CSPG were off-target, *i.e.* mediated by effects on a target other than ErbB1, then PD168393 should enhance neurite outgrowth on CSPG even in ErbB1 −/− cultures. In fact, neurite outgrowth from ErbB1 −/− cerebellar granule cells and DRG neurons on CSPG was not improved by PD168393 (open bars in Figs. 3A, C).

**Supplementary Fig. 2.**
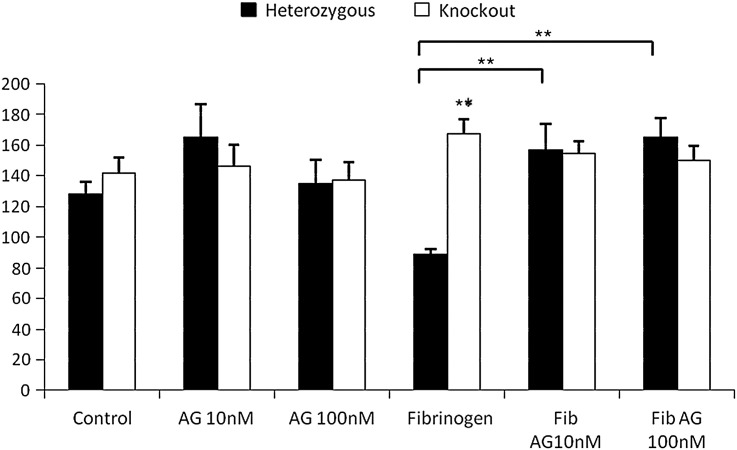
Cerebellar granule cells were cultured on a control substrate in the presence of fibrinogen and/or AG1478 at the indicated concentrations. N = 6 Two way ANOVA *post hoc* Bonferroni, ** = p < 0.01 when compared to heterozygous cells under the same culture conditions unless shown otherwise.

**Fig. 4:**
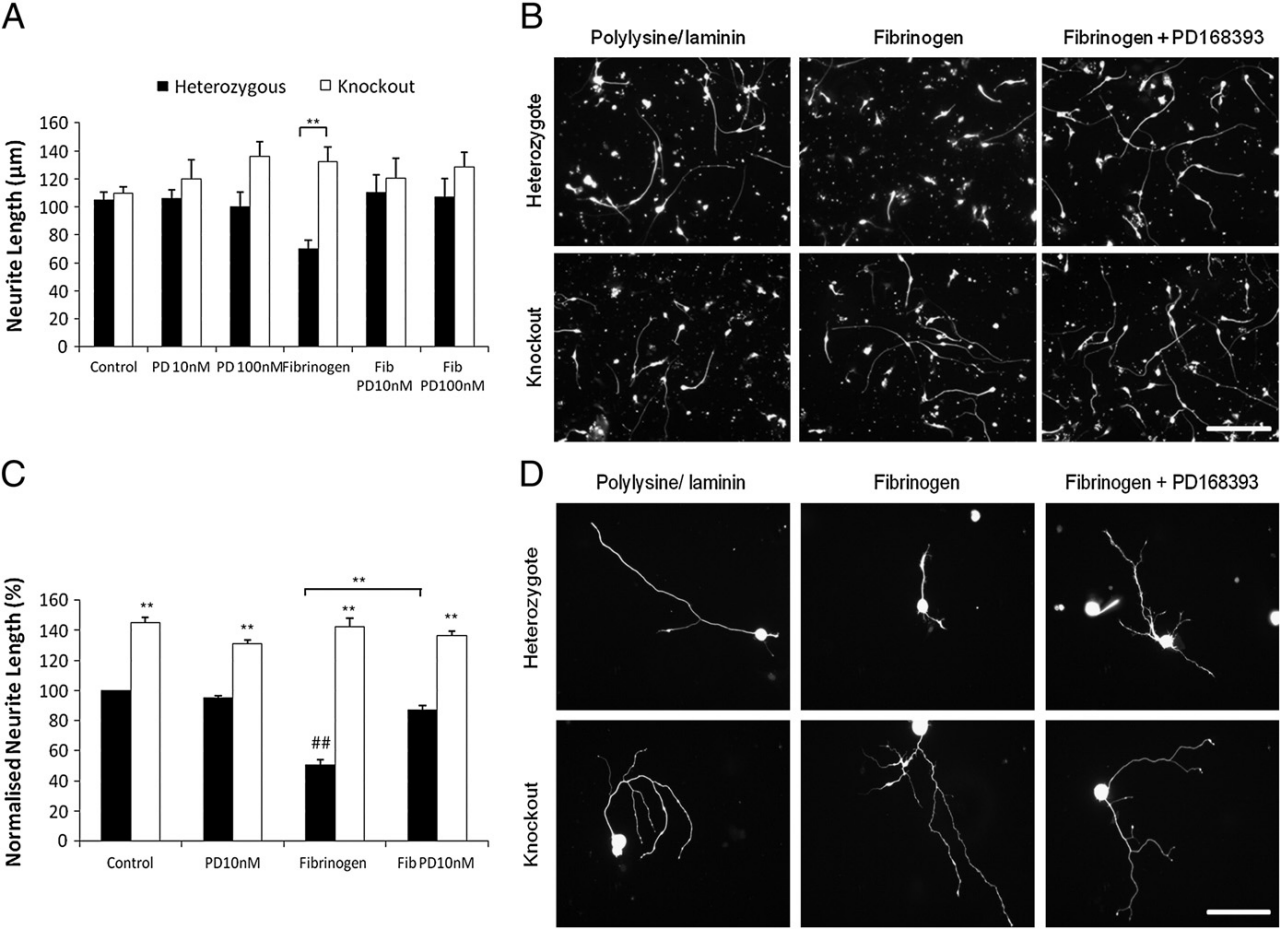
PD168393 rescues outgrowth in the presence of fibrinogen from ErbB1 expressing neurons but does not affect ErbB1 −/− neurons. A: Cerebellar granule cells were cultured on a control substrate in the presence of fibrinogen and/or PD168393 at the indicated concentrations. N = 6. Two way ANOVA *post hoc* Bonferroni, ** = p < 0.01. B: Representative images of cultured cerebellar granule neurons from an ErbB1 −/− pup and heterozygote littermates on polylysine/laminin substrates with or without fibrinogen fixed and stained for neuron-specific β3 tubulin. The ErbB1 kinase inhibitor PD168393 was present at 10 nM where indicated. Images were taken at × 20 magnification and scale bar equals 100 μm. The scale bar applies to all panels. C: Sensory neurons were cultured on a control substrate in the presence of fibrinogen and/or PD168393 at the indicated concentrations. Neurite length was normalised to the control heterozygous measurement. N = 6. Two way ANOVA *post hoc* Bonferroni ** = p < 0.01 when compared to heterozygous cells under the same culture conditions unless shown otherwise, ^##^ = p < 0.01 when compared to neurons of the same genetic background on a control substrate. D: Representative images of cultured sensory neurons from an ErbB1 −/− pup and heterozygote littermates on polylysine/laminin substrates with or without fibrinogen fixed and stained for neuron-specific β3 tubulin. The ErbB1 kinase inhibitor PD168393 was present at 10 nM where indicated. Images were taken at × 20 magnification and scale bar equals 100 μm. The scale bar applies to all panels.

The blood–brain barrier normally precludes contact of CNS neurons with the blood protein fibrinogen, but at injury sites fibrinogen permeates into the central nervous system tissue. Fibrinogen inhibits neurite outgrowth from sympathetic neurons and cerebellar granule neurons, and this outgrowth can be rescued by PD168393 (Schachtrup et al., 2007). We confirmed this finding for ErbB1-expressing sensory neurons and cerebellar granule neurons (Fig. 4). In contrast, neurite outgrowth from ErbB1 −/− neurons of either type was unaffected by fibrinogen at 0.5 μg/ml (Fig. 4) (although fibrinogen at 1.5 μg/ml gave a slight but statistically significant inhibition, data not shown). The reversible ErbB1 inhibitor AG1478 has been reported, like PD168393, to rescue neurite outgrowth on inhibitory substrates (Douglas et al., 2009; Koprivica et al., 2005). AG1478 rescued outgrowth from ErbB1-expressing neurons in the presence of fibrinogen but was without effect on ErbB1 −/− neurons (Supplemental Fig. 2). Hence the genetic deletion of ErbB1 disinhibits neurite outgrowth from both cerebellar granule cells and primary sensory neurons cultured in the presence of fibrinogen. AG1478 also disinhibited neurite outgrowth through effects on ErbB1. Together, these findings show that three of the most important factors inhibiting neurite outgrowth at lesion sites in the central nervous system exert much of their effect through ErbB1, and that all the rescue of outgrowth seen when the drugs PD168393 or AG1478 are applied is due to the effect of these drugs in blocking the action of ErbB1.

### ErbB1 and calcium signalling are involved in the inhibition of axonal outgrowth by synthetic double stranded RNA

**Fig. 5:**
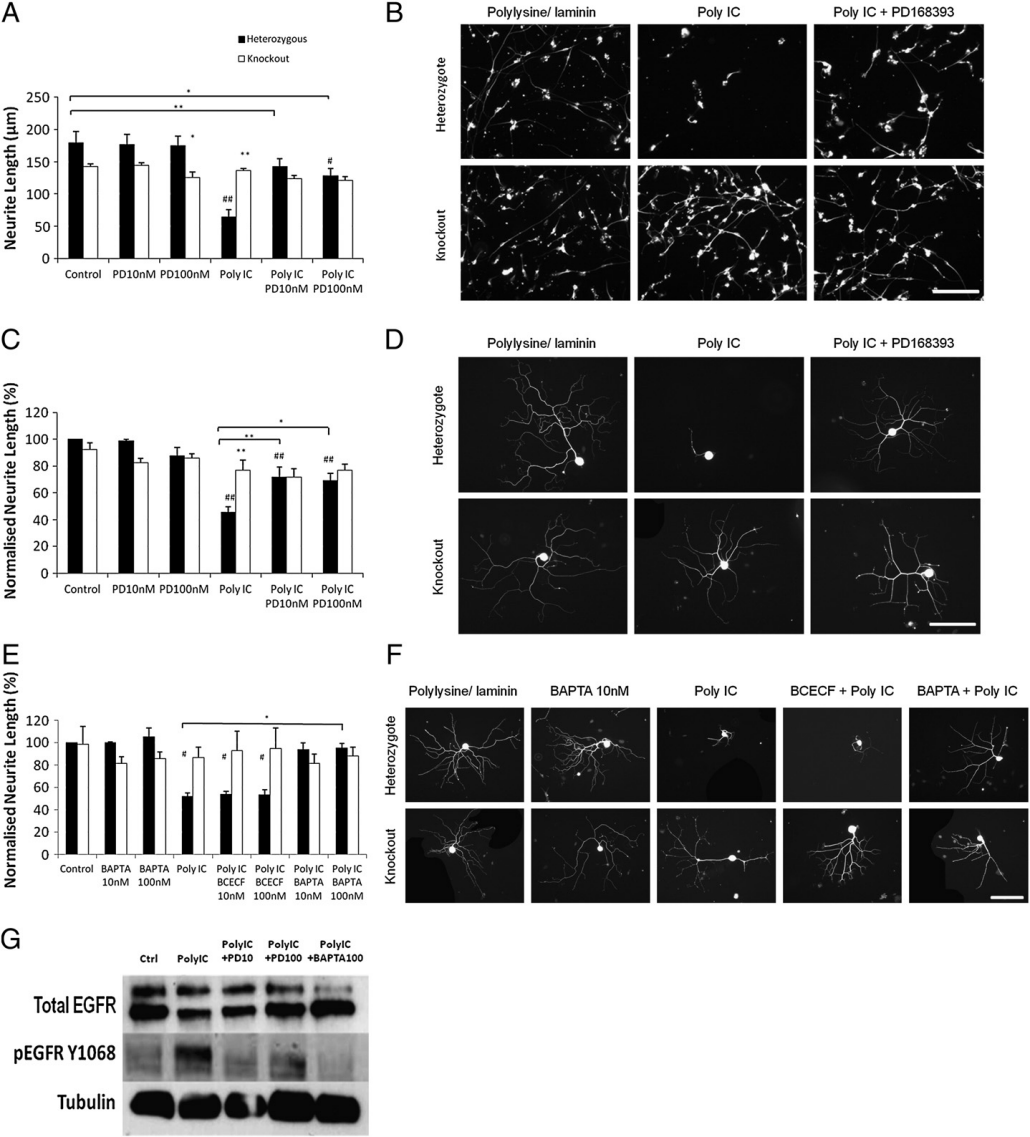
The TLR3 agonist poly I:C inhibits outgrowth by an ErbB1- and calcium-dependent mechanism. A: Heterozygous and ErbB1 −/− cerebellar granule neurons were cultured on a control substrate in the presence of PD168393 and/or poly I:C at the indicated concentrations. N = 8. Two way ANOVA *post hoc* Bonferroni, ** = p < 0.01 * = p < 0.05 when compared to heterozygous cells under the same conditions unless shown otherwise, ^##^ = p < 0.01 ^#^ = p < 0.05 when compared to genetically identical cells under control conditions. B: Representative images of cultured cerebellar granule neurons from an ErbB1 −/− pup and heterozygote littermates on polylysine/laminin substrates with or without poly I:C then fixed and stained for neuron-specific β3 tubulin. The ErbB1 kinase inhibitor PD168393 was present at 10 nM where indicated. Images were taken at × 20 magnification and scale bar equals 100 μm. The scale bar applies to all panels. C: Heterozygous and ErbB1 −/− sensory neurons were cultured on a control substrate in the presence of PD168393 and/or poly I:C at the indicated concentrations. Neurite length was normalised to the control heterozygous measurement. N = 5. Two way ANOVA *post hoc* Bonferroni ** = p < 0.01 * = p < 0.05 when compared to heterozygous cells under the same culture conditions unless shown otherwise, ^##^ = p < 0.01 when compared to genetically identical cells under control conditions. D: Representative images of cultured sensory neurons from an ErbB1 −/− pup and heterozygote littermates on polylysine/laminin substrates with or without poly I:C fixed and stained for neuron-specific β3 tubulin. The ErbB1 kinase inhibitor PD168393 was present at 10 nM where indicated. Images were taken at × 20 magnification and scale bar equals 100 μm. The scale bar applies to all panels. E: Heterozygous and ErbB1 −/− sensory neurons were cultured on a control substrate in the presence of PD168393 and/or BCECF AM or BAPTA AM at at the indicated concentrations. Neurite length was normalised to the control heterozygous measurement. N = 5. Two way ANOVA *post hoc* Bonferroni * = p < 0.05, ^#^ = p < 0.05 when compared to genetically identical cells under control conditions. F: Representative images of cultured sensory neurons from an ErbB1 −/− pup and heterozygote littermates on polylysine/laminin substrates in the presence of PD168393 and/or BCECF AM or BAPTA AM at 10 nM, fixed and stained for neuron-specific β3 tubulin. Images were taken at × 20 magnification and scale bar equals 100 μm. The scale bar applies to all panels. G: Western blot showing the effects of 16 h treatment with Poly I:C, PD168393 and BAPTA on ErbB1 kinase activity in cultured cerebellar granule cells. Transfer membranes were probed with an antibody specific for ErbB1 phosphorylated on Y1068.

Viral infections during pregnancy are now believed to be important sources of brain abnormalities and subsequent behavioural problems. Much of this effect is due to activation by double stranded viral RNA of Toll-like receptor 3 (TLR3) on both neurons and immune system cells as part of the defence mechanism against viral infections (Brown and Derkits, 2010; Daffis et al., 2008; De Miranda et al., 2010; Meyer et al., 2008; Takeda and Akira, 2007). Activators of TLR3, either double stranded RNA itself or the artificial analogue poly I:C, have been reported to be potent inhibitors of neurite outgrowth from embryonic mouse or chick sensory neurons (Cameron et al., 2007). Since TLR3 exerts at least part of its effect in non-neuronal cells via activation of ErbB1 (Koff et al., 2008) we examined whether the inhibitory effect on neurite outgrowth, like that of myelin, CSPG and fibrinogen, acted through ErbB1. We first confirmed that poly I:C is a potent inhibitor of axonal regeneration *in vitro*. Measurements of neurite outgrowth showed a very similar pattern with poly I:C as with CSPG and fibrinogen: ErbB1-expressing neurons were inhibited by poly I:C at 100 μg/ml and rescued by PD168393, whilst ErbB1 −/− neurons are unaffected by poly I:C (Figs. 5A–D). Once again, the absence of effects of PD168393 on ErbB1 −/− cultures showed that off-target effects were not involved. Buffering of intracellular calcium changes was reported to prevent the activation of ErbB1 otherwise affected in cerebellar granule cells by exposure to myelin (Koprivica et al., 2005). To examine whether the ErbB1- dependent inhibition of neurite outgrowth by poly I:C is similarly dependent on intracellular calcium signals, we performed outgrowth assays in the presence of the cell permeable calcium buffer BAPTA-AM. Significant rescue of neurite outgrowth from ErbB1-expressing sensory neurons was seen in the presence of 100 nM BAPTA-AM (solid bars in Fig. 5E). In contrast BCECF-AM, a pH buffer which utilises the same esterase reactions to enter cells, was without effect. As expected, BAPTA-AM did not improve neurite outgrowth from ErbB1 −/− neurons on any substrate (open bars in Fig. 5E).

The inhibitory effect of all the outgrowth-inhibitory factors studied is much more marked in cultures of cells expressing ErbB1 than in ErbB1 −/− cultures. Indeed, fibrinogen at 0.5 μg/ml, and poly I:C, had no detectable inhibitory effect on outgrowth from ErbB1 −/− cerebellar granule neurons and sensory neurons, whilst CSPG had no detectable inhibitory effect on outgrowth from ErbB1 −/− sensory neurons. Myelin, which contains multiple inhibitory factors, inhibited neurite outgrowth from ErbB1 −/− neurons of both types, although not as strongly as from ErbB1 expressing neurons (Fig. 2; neurite length of DRG cells on myelin was 28 ± 4% and 52 ± 11% of the length on polylysine/laminin for +/− and −/− neurons respectively, N = 5). Our results strongly support the hypothesis that multiple inhibitors of neurite outgrowth act through the ErbB1 epidermal growth factor receptor (Koprivica et al., 2005; Schachtrup et al., 2007).

## Discussion

ErbB1 inhibitors offer a real hope of improving the regeneration of axons in the injured CNS, but the role of ErbB1 in mediating the inhibition of neurite outgrowth has been challenged (Douglas et al., 2009). We have used neurons from mice lacking ErbB1 to address this question and have established unequivocally that ErbB1 is a critical mediator for a key group of cues that prevent axonal regeneration in the injured brain and spinal cord: CNS myelin, CSPGs and fibrinogen. In contrast to a previous hypothesis (Douglas et al., 2009), we show that the ErbB1 kinase inhibitors PD168393 and AG1478 rescue neurite outgrowth through their action on ErbB1 itself. We have also shown for the first time that the Toll-like receptor 3 agonist and viral mimic poly I:C also inhibits axonal regeneration through a pathway involving calcium signalling upstream of ErbB1 activation.

The failure of axonal regeneration in the CNS of adult mammals is believed to be partly the result of the presence of multiple inhibitors of axonal growth both within CNS tissue and in and around lesion sites in the CNS (Anderson et al., 2007). This is an attractive hypothesis, not least because it should be possible to pharmacologically block the receptors of such inhibitors. Unfortunately, blocking or genetically deleting the known receptors for myelin proteins and CSPGs, or blocking or eliminating specific inhibitors, despite encouraging early studies (GrandPre et al., 2002; Li et al., 2005), has generally failed to produce reliable regeneration of CNS tracts (Steward et al., 2008; Zheng et al., 2005). RhoA appeared to be an important target whose inhibition should block several axonal growth inhibitors but although RhoA inhibitors have also been found in experiments by several laboratories to promote axonal regeneration *in vivo*, their efficacy is relatively modest (Bertrand et al., 2005; Boato et al., 2010; Fournier et al., 2003). It is therefore important to identify further targets for pharmacological intervention to promote axonal regeneration following CNS injury.

Viral infections during pregnancy are now believed to be important sources of brain abnormalities (De Miranda et al., 2010; Meyer et al., 2008) and subsequent behavioural problems (Brown and Derkits, 2010). Our findings indicate that the viral mimic poly I:C, an artificial double stranded RNA, inhibits axonal elongation via calcium signalling and ErbB1. This may help in the identification of targets for interventions to limit the effects of viral infections on the developing nervous system. In addition, TLR3 may be activated by RNA released from damaged mammalian cells (Kariko et al., 2005), which thereby constitute another potentially inhibitory influence at CNS lesion sites, mediating at least some of its effects through ErbB1.

Although it is agreed that ErbB1 antagonists promote axonal growth in the presence of otherwise inhibitory molecules (Koprivica et al., 2005), two recent papers have suggested that the effect of the anti-ErbB1 agents PD168393 and AG1478 in rescuing neurite outgrowth on inhibitory substrates is due to their acting off-target rather than directly on ErbB1 (Ahmed et al., 2009; Douglas et al., 2009). Our results are incompatible with this view, since neither PD168393 nor AG1478 improved neurite outgrowth from ErbB1 −/− neurons under any conditions. PD168393 and AG1478 were only able to improve neurite outgrowth in cultures expressing ErbB1, confirming that this receptor is their critical site of action. It might be argued that, since in a number of cases, inhibitory molecules failed to reduce neurite outgrowth from ErbB1 −/− neurons, the fact that PD169393 and AG1478 did not then improve neurite outgrowth is not evidence that these agents are acting through ErbB1 and not off-target. However, even under conditions where ErbB1 −/− neurons experienced a significant residual inhibition (cerebellar granule cells on CSPGs and myelin; sensory neurons on myelin) PD169393 completely failed to rescue neurite outgrowth. The most likely explanation of the apparent discrepancy between our results and those in the earlier papers (Ahmed et al., 2009; Douglas et al., 2009) is that siRNA knockdown of ErbB1, used in the previous studies, does not eliminate sufficient ErbB1 to prevent its function in limiting axonal regeneration *in vitro*.

Myelin proteins and CSPG are believed to be among the dominant inhibitors of axonal regeneration operating around and within the central nervous system lesion sites (Anderson et al., 2007; Sandvig et al., 2004). It is therefore important to note that not all the inhibitory effects of these agents can be attributed to ErbB1 activation. Both agents retained significant inhibitory activity in cultures lacking ErbB1. In cultures expressing ErbB1, PD168393 was able to reverse all the ErbB1-dependent component of inhibition, but the ErbB1-independent component of inhibition remained (*e.g.* Figs. 2A, 3A). This is not surprising in view of the multiplicity of receptors involved (Anderson et al., 2007). Ablating the known receptors for CSPGs does not eliminate all inhibition mediated by those molecules (Dickendesher et al., 2012; Shen et al., 2009). In addition, CNS myelin is known to contain several inhibitors of axonal outgrowth including Sema 4D (Moreau-Fauvarque et al., 2003). Semaphorins do not act through calcium signalling and ErbB1 phosphorylation (Koprivica et al., 2005; Wen et al., 2004). Thus ErbB1 inhibitors are likely to prove a component of a therapy for enhancing axonal regeneration following CNS injury rather than a stand-alone treatment.

It is not yet clear how ErbB1 activation is brought about by CNS myelin, fibrinogen, CSPGs or poly I:C or how ErbB1 activation reduces the ability of neurons to extend their axons. Autophosphorylation of ErbB1 promotes cell survival and division in non-neuronal cells but more recent work has shown that ErbB1 is the hub of a complex web of inputs and outputs. Direct activating ligands include TGFα, heparin-binding EGF (HB-EGF), betacellulin, amphiregulin, epiregulin and SOCS2 (Adlkofer and Lai, 2000; Goldshmit et al., 2004) whilst transactivation pathways allow G protein coupled receptors such as the muscarinic acetylcholine receptor to activate ErbB1 (Zwick et al., 1999). Less well understood pathways allow yet other classes of extracellular ligands to activate ErbB1; for example components of the extracellular matrix act through integrins to activate ErbB1 (Cabodi et al., 2004). There is longstanding evidence that calcium is important in the signalling from CNS myelin to bring about cessation of axonal growth (Bandtlow et al., 1993; Loschinger et al., 1997). Koprivica et al. (2005) found that calcium signalling was upstream of ErbB1 activation by myelin and CSPGs. We have shown that calcium signalling is necessary for ErbB1 activation and the inhibition of neurite outgrowth by poly I:C in cultures expressing ErbB1. Taken together, these data suggest a general mechanism in which calcium signals, elicited by a wide range of cues (Gomez and Spitzer, 1999; Gomez and Zheng, 2006; Lautermilch and Spitzer, 2000; Zheng and Poo, 2007), act *via* ErbB1 to inhibit axon growth.

Once activated, ErbB1 has an enormous range of intracellular targets in addition to the classical MAP kinase and protein kinase B pathways, and can translocate to either the nucleus or mitochondria to act upon substrates there (Demory et al., 2009; Lo, 2010). Although much remains to be discovered about the mechanism of action of ErbB1 on axonal regeneration our results show that ErbB1 is a negative regulator of axonal outgrowth, they show that ErbB1 inhibitors act directly on their primary target to enhance axonal regeneration, and they indicate that ErbB1 inhibitors may prove an important component of multivalent strategies for the restorative treatment of central nervous system injury. Furthermore, the considerable knowledge of ErbB1 signalling pathways gleaned from studies of cancer biology can now be applied to enhancing axonal regeneration following spinal cord injury.

## Role of funding source

This work was supported by the Wellcome Trust [grant number 087548].

The following are the supplementary data related to this article.
